# The potential benefit of leptin therapy against amyotrophic lateral sclerosis (ALS)

**DOI:** 10.1002/brb3.2465

**Published:** 2021-12-21

**Authors:** Agueda Ferrer‐Donato, Ana Contreras, Paloma Fernandez, Carmen M. Fernandez‐Martos

**Affiliations:** ^1^ Neurometabolism Research Lab., Hospital Nacional de Paraplejicos SESCAM Toledo Spain; ^2^ Centro de Investigación en Salud (CEINSA) Universidad de Almería Almería Spain; ^3^ Institute of Applied Molecular Medicine (IMMA) Faculty of Medicine Universidad San Pablo CEU Madrid Spain; ^4^ Wicking Dementia Research and Education Centre College of Health and Medicine University of Tasmania Hobart Australia

**Keywords:** amyotrophic lateral sclerosis (ALS), TAR DNA binding protein (TDP‐43)

## Abstract

**Background:**

Targeting leptin could represent a rational strategy to treat amyotrophic lateral sclerosis (ALS), as previously clinical studies have shown its levels to be associated with a lower risk of ALS disease. However, very little is known about the potential influence of leptin in altering disease progression in ALS, as it has thus far been correlated with the protection exerted by increased fat mass stores.

**Methods:**

We studied the impact of leptin treatment beginning at 42‐days of age (asymptomatic stage of disease) in the TDP‐43 (TDP43^A315T^) transgenic (Tg) ALS mouse model.

**Results:**

Our study shows that leptin treatment was associated with altered expression of adipokines and metabolic proteins in TDP43^A315T^ mice. We also observed that weight loss decline was less prominent after leptin treatment in TDP43^A315T^ mice relative to vehicle‐treated animals. In TDP43^A315T^ mice treated with leptin the disease duration lasted longer along with an improvement in motor performance relative to vehicle‐treated animals.

**Conclusions:**

Collectively, our results support leptin as a potential novel treatment approach for ALS.

## Abstract 2

There is an urgent need to improve upon current approaches to amyotrophic lateral sclerosis (ALS). In this study, we evaluated in vivo the impact of leptin treatment in a very well validated mouse model of TDP‐43 proteinopathy (TDP‐43A315T mice). Collectively, our results support leptin as a potential novel treatment approach for ALS.

## 1 INTRODUCTION

Amyotrophic lateral sclerosis (ALS) is a motor neuron disease (MND) characterized by the selective and progressive loss of upper and lower motor neurons of the cerebral cortex, brainstem, and the spinal cord (Tapia, 2014). The estimated prevalence of this fatal neurological disorder is 5 per 100,000 in the United States, and approximately 2–3 people per 100,000 of the general population in Europe (Barcelo et al., 2021). Over 60% of patients die within 3–5 years of diagnosis. The majority of patients have sporadic ALS (sALS) (more than 90%) in which multiple risk factors from gene‐environment interactions contribute to the disease pathogenesis. In contrast, only a small subset of patients has familiar ALS (fALS) (less than 10%) due to their associated genetic dominant inheritance factor (Zarei et al., 2015). The putative mechanisms by which mutations or gene‐environment interactions cause the progressive degeneration and death of motor neurons have not been completely elicited yet. There have been an overwhelming number of experimental studies during the last few years providing insights into the pathogenesis of ALS and revealing the complexity and heterogeneity of ALS; however, these have not provided an efficacious drug for this condition. ALS therefore represents one of the most challenging socio‐economic problems of our future. There is an urgent need to improve upon current approaches to ALS.

Leptin is a polypeptide hormone primarily secreted by adipocytes that exerts its main biological function in the brain (Stephens et al., 1995; Zhang et al., 1994). Leptin acts by binding to receptors that are structurally related to the cytokine receptor class I family, while only its long isoform (Ob‐Rb) is thought to transmit the majority of leptin's biological signals (Friedman & Halaas, 1998). However, in addition to its classical role in the neuroendocrine regulation of food intake, both clinical and epidemiological data show leptin to be a promising neuroprotective drug for progressive neurological conditions. Accumulating in vivo and in vitro studies have suggested that leptin has notable effects on neuroprotection (Bahor et al., 2017; Begley & Ellis, 2012; Fernandez‐Martos et al., 2017; Liu et al., 2020; Marwarha et al., 2010) as well as on improving learning and cognitive function in Alzheimer´s disease (AD) (Farr et al., 2006; Sato et al., 2011; Searcy et al., 2012). Many of the neurological beneficial properties of leptin are currently being experimentally probed in order to get a strong foothold of the therapeutic implications for other neurological disorders as Parkinson's disease (Rahnemayan et al., 2021; Zou et al., 2019). In the context of ALS, leptin levels are inversely associated with ALS risk (Nagel et al., 2017). A positive correlation of plasma leptin and body mass index (BMI) was observed in ALS patients (Ngo et al., 2015). This is of interest because patients with ALS are unable to maintain their body weight (Bouteloup et al., 2009; Desport et al., 2001), and rapid weight loss is clinically associated with worse disease outcomes (Ahmed et al., 2019; Kuraszkiewicz et al., 2020). Indeed, weight loss is a predictor of shorter survival in ALS (Lee et al., 2021), raising the possibility that leptin could be exploited to derive therapeutic effects.

Given the evidence showing leptin levels related with overall survival and prognosis of ALS patients (Nagel et al., 2017), this study aimed to determine the impact of leptin treatment in the well validated TAR DNA binding protein (TDP‐43) A315T murine model of ALS TDP‐43 proteinopathy (Hatzipetros et al., 2014; Wegorzewska et al., 2009), which recapitulates several aspects of the human ALS, providing, to our knowledge, the first insights into the potential benefit of leptin to ALS.

## 2 MATERIAL AND METHODS

### 2.1 Animals

TDP43^A315T^ mice (Hatzipetros et al., 2014; Wegorzewska et al., 2009) and age and gender‐matched wildtype (WT) littermate controls (Strain No. 010700, Bar Harbor, ME, USA) were used in this study. This mouse model of ALS expresses a mutant human TDP‐43 (hTDP‐43) cDNA harboring an N‐terminal Flag tag and an A315T amino acid substitution associated with ALS mainly in the CNS (Wegorzewska et al., 2009). To avoid ambiguity associated with reported sex‐related differences in mean survival time of TDP‐43^A315T^ mice (Hatzipetros et al., 2014; Wegorzewska et al., 2009), only male mice were used. Animals expressing the hTDP‐43 transgene were confirmed via polymerase chain reaction according to the distributor's protocol. Animals were group‐housed under standard housing conditions with a 12 h light–dark cycle, and food and water ad libitum. To monitor disease progression and onset determination, all mice were weighed and assessed three times per week until the disease onset‐stage, after which they were checked daily in the morning until the disease end‐stage. All experimental procedures were approved by the Animal Ethics Committee of the National Hospital for Paraplegics (HNP) (Approval No 26/OH 2018) in accordance with the Spanish Guidelines for the Care and Use of Animals for Scientific Purposes.

### 2.2 Leptin treatment

Both TDP‐43^A315T^ mice and WT littermate controls were divided into two subgroups (*n* = 3–5 Tg mice/ subgroup and *n* = 4 WT mice/ subgroup) according to the treatments. Beginning at 42 days of age animals were treated with 0.03 mg/kg/day intranasal (IN) recombinant mouse leptin (Sigma Aldrich) (Fliedner et al., 2006) or vehicle (VH) daily for 14 consecutive days, because 14 days of treatment with leptin has been demonstrated to be neuroprotective in vivo under degenerative conditions in the APP/PS1 (APPswe/PSEN1dE9) Tg AD line (Fernandez‐Martos et al., 2017; Liu et al., 2020). Recombinant mouse leptin was prepared in 0.125% (2.3 mm) of *N*‐tetradecyl‐*b*‐d‐maltoside (TDM) (Sigma‐Aldrich) reconstituted in phosphate buffer saline (PBS; pH 7.2) at a concentration of 1 mg/ml. The alkylglycoside, TDM, was used to increase the bioavailability of leptin for IN administration (Arnold et al., 2004). IN administration was conducted as previously described (Fernandez‐Martos et al., 2017). Control groups of each genotype received the IN VH solution (PBS‐TDM; pH 7.2) daily for 14 consecutive days. Mice were monitored closely in terms of their mobility or level of activity immediately after the procedure. No differences in weight gain between groups (Lep‐treated vs. VH‐treated) were displayed (data not shown). All analyses were conducted by personnel blinded to the animal genotype.

### 2.3 Monitoring and behavioral assessments

To monitor disease progression and onset (defined as the last day of individual peak body weight before gradual loss occurs) determination, body weight lost was measured and motor performance was evaluated using rotarod test. All mice were weighed and assessed three times per week until the disease onset‐stage. After that mice were then checked daily in the morning until the disease end‐stage (defined as the weight below 20% of the initial weight on each of 3 consecutive days). The rotarod motor test was performed on all mice once a week (Dang et al., 2014), starting from the 7 weeks of age until the day of euthanasia. Animals were previously trained for three consecutive days and three times a day to promote the learning of the task. The accelerated protocol was applied for this motor monitoring as described previously by Mandillo et al. (Mandillo et al., 2008). In brief, mice were placed on a rotarod apparatus (Model 7650, Ugo Basile) at a speed of 4 rpm with acceleration up to 40 rpm over 300 s. Three tests were performed for each mouse with a minimal interval of 20 min, and the average of the longest two performances was taken as the final result for analysis.

### 2.4 Sample preparation

At disease end‐stage, animals were terminally anesthetized with sodium pentobarbitone (140 mg/kg) and transcardially perfused with room temperature 0.01 m phosphate buffered saline (PBS; pH 7.4). Blood was collected and processed as previously described (Rodriguez et al., 2021). Plasma samples were immediately frozen on dry ice and stored at –80°C for later analysis.

### 2.5 Measurement of metabolic markers in plasma

Total ghrelin, the adipokines, resistin, and leptin, and metabolic biomarkers of insulin resistance (GIP, GLP‐1, glucagon, PAI‐1, and insulin) from plasma samples were analyzed by duplicate using the Bio‐PlexPro mouse Diabetes group from Bio‐Rad (Ref. 171F7001M) by Luminex^®^ 200™ technology as previously described (Ortega Moreno et al., 2020). Samples were processed following the manufacturer's instructions. According to Bio‐Rad's information the intra‐ and inter‐assay CV variability is < 20%. The final concentration value of each metabolic marker was the result of the mean from the two duplicated measures.

### 2.6 Statistical analysis

All data are presented as means ± standard error of the mean (SEM). Differences between means were assessed by two‐way ANOVA followed by Dunett's post hoc test, to compare all groups with VH‐treated WT mice, and Tukey's post hoc test was used for multiple comparisons between all groups. For multiplex assays, the mean of each experimental group was determined for all the analytes, and Kruskal–Wallis test was performed followed by Dunett's post hoc test to compare all groups with onset stage, while Bonferroni post hoc test was used for multiple comparisons between all groups. For all statistical tests, a *p* value of < .05 (CI 95%) was assumed to be significant. Statistical analysis was performed using GraphPad Prism software (version 8.3.1).

## 3 RESULTS

### 3.1 Leptin treatment modifies disease progression and improves motor performance in TDP‐43^A315T^ mice

**FIGURE 1:**
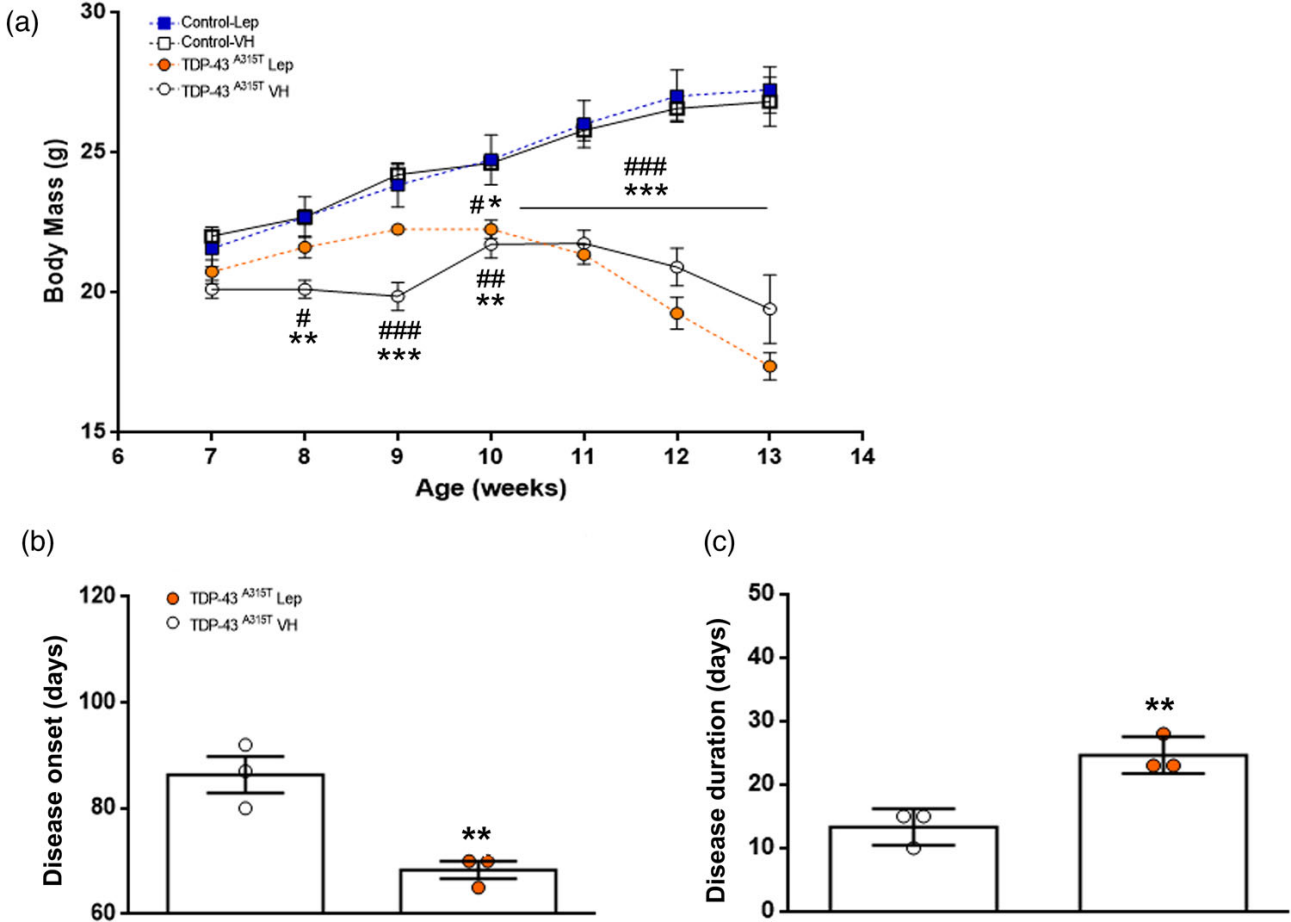
Leptin treatment alters body weight and changes disease onset and duration in TDP‐43^A315T^ mice. (a) Body weight was monitored over time in WT controls and TDP‐43^A315T^ mice treated with 0.03 mg/kg/day IN with leptin or VH (PBS‐TDM; pH 7.2) daily for 14 consecutive days, beginning at the asymptomatic state, at 42 days of age. Starting weight on week 7. Although there was a trend, no significant differences were observed between Lep‐treated TDP‐43^A315T^ mice or VH‐treated TDP‐43^A315T^ mice. (b) Average disease onset and (c) disease duration were determined in WT controls and TDP‐43^A315T^ mice treated with leptin or VH using body weight as a physiological parameter. Average disease duration of the animal was calculated as the time between the onset of disease (defined as the last day of individual peak bodyweight before gradual loss occurs) and the day of death. Comparatively the disease duration was higher in Lep‐treated TDP‐43^A315T^ mice vs. VH‐treated TDP‐43^A315T^ mice. Values are expressed as mean ± SEM. Comparison between groups was performed by two‐way ANOVA followed by Dunett's post hoc test to compare all groups with VH‐treated WT mice, while Tukey's post hoc test was used for multiple comparisons between all groups, where (a) * *p* < .05, ** *p* < .01, *** *p* < .001  versus VH‐treated WT mice; # *p* < .05, ## *p* < .01, ### *p* < 0.001 versus Lep‐treated WT mice; (b) ** *p* < .01  versus VH‐treated TDP‐43^A315T^ mice; and (c) ** *p* < .01  versus Lep‐treated TDP‐43^A315T^ mice. Corresponding graphs as per (a), that is, VH‐treated WT mice (*n*  =  4, white square and solid line), Lep‐treated WT mice (*n*  =  4, blue square and dashed line), VH‐treated TDP‐43^A315T^ mice (*n*  =  3, white circles and solid line), and Lep‐treated TDP‐43^A315T^ mice (*n*  =  3, orange circles and dashed line)

The biological impact of leptin treatment during the progression of ALS is unknown, even though this hormone has previously been shown to be associated with a lower risk of ALS disease and to confer a survival advantage in ALS patients (Nagel et al., 2017; Ngo et al., 2015). Thus, as it have been previously reported that TDP‐43^A315T^ mice exhibit weight loss during disease progression (Esmaeili et al., 2013; Guo et al., 2012; Hatzipetros et al., 2014; Medina et al., 2014; Rodriguez et al., 2021), we assessed the capacity of leptin treatment to modify weight changes over time in TDP‐43^A315T^ mice compared to age‐matched WT littermates (Figure 1). A two‐way ANOVA with repeated measures revealed a significant genotype interaction (Figure 1a; F_(18, 83) _= 7.701, *p* < .0001), indicating a sustained decline in body weight in TDP‐43^A315T^ mice compared to WT in response to leptin treatment over time. Although there was a trend, no significant difference was found between Lep‐treated or VH‐treated TDP‐43^A315T^ mice, while weight loss decline seemed less prominent in Lep‐treated TDP‐43^A315T^ mice until week 11 (Figure 1a). In addition, the calculation of the disease onset using this parameter indicated that Lep‐treated TDP‐43^A315T^ mice develop symptoms earlier than VH‐treated TDP‐43^A315T^ mice (Figure 1b; *t*_5 _= 5.576 *p* < .01). Using body weight measurements (criterion of 10% body weight loss), an average disease onset of 86 ± 2.5 days of age was determined in VH‐treated TDP‐43^A315T^ mice, whereas Lep‐treated TDP‐43^A315T^ mice presented a phenotype at 68 ± 1.7 days of age (Figure 1b). Indeed, when analyzing disease duration, calculated as the time between disease onset and disease end‐stage, our data showed a longer disease duration in TDP‐43^A315T^ mice after leptin treatment compared to VH treatment (Figure 1c; *t*_4 _= 4.808 *p* < .01).

**FIGURE 2:**
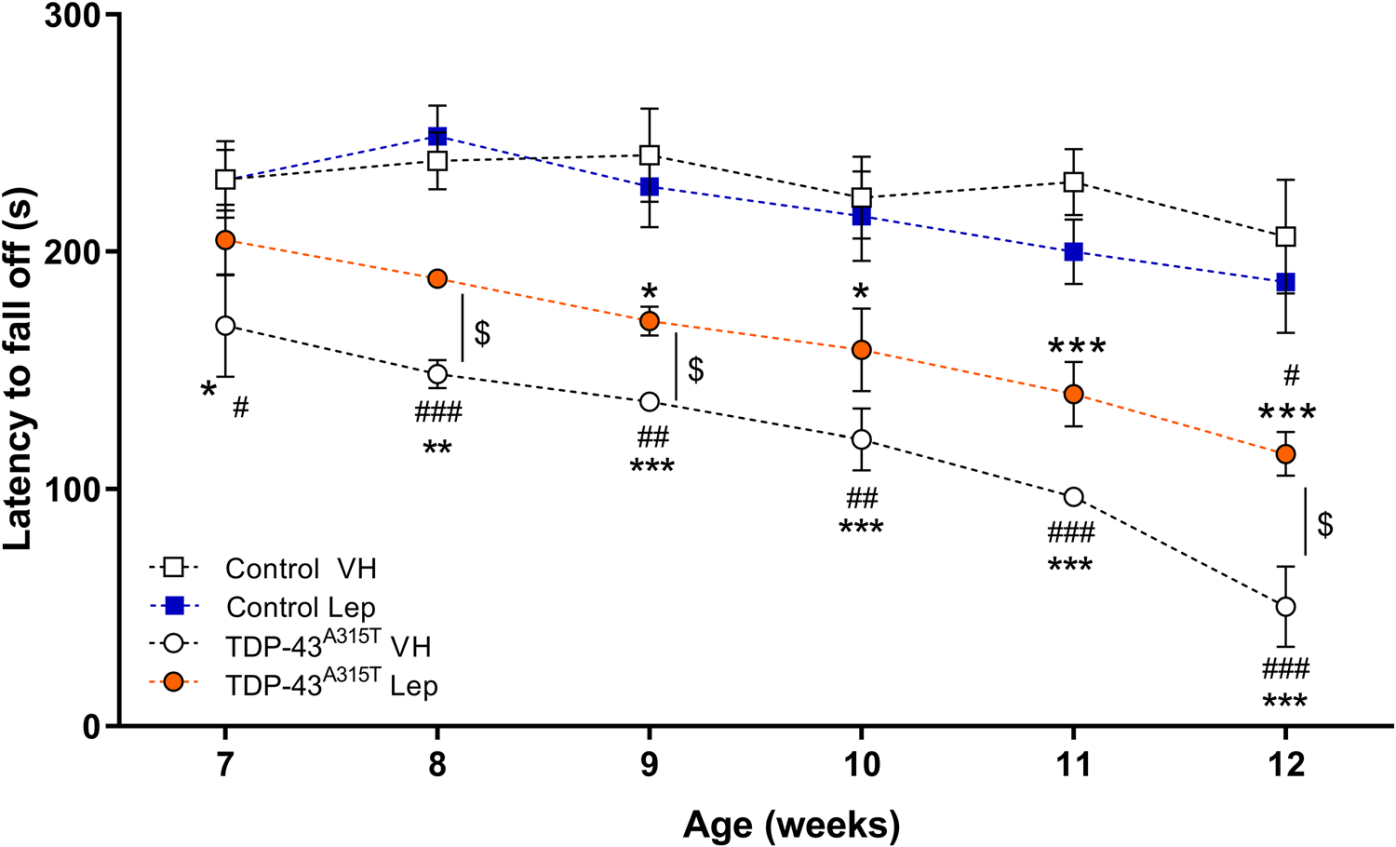
Leptin treatment significantly improves motor performance in TDP‐43^A315T^ mice. Behavioral assessment of motor function was performed in WT controls and TDP‐43^A315T^ mice treated with leptin or VH over time. Significant differences between Lep‐ and VH‐treated mice were seen. Values are expressed as mean ± SEM. Comparison between groups was performed by two‐way ANOVA followed by Dunett's post hoc test to compare all groups with VH‐treated WT mice, while Tukey's post hoc test was used for multiple comparisons between all groups, where * *p* < .05, ** *p* < .01, *** *p* < .001 versus VH‐treated WT mice; # *p* < .05, ## *p* < .01, ### *p* < .001 1 versus Lep‐treated WT mice; $ *p* < .05 versus VH‐treated TDP‐43^A315T^ mice. Corresponding graphs, VH‐treated WT mice (*n*  =  4, white square and solid line), Lep‐treated WT mice (*n*  =  4, blue square and dashed line), VH‐treated TDP‐43^A315T^ mice (*n*  =  3, white circles and solid line), and Lep‐treated TDP‐43^A315T^ mice (*n*  =  3, orange circles and dashed line)

Moreover, we also tested motor behavior to determine if leptin treatment could alter motor‐disease phenotype. Our results indicate a progressive decline in motor coordination in TDP‐43^A315T^ mice (Figure 2), confirming the progressive motor deficits of the TDP‐43^A315T^ mouse model. A two‐way ANOVA with repeated measures revealed a significant effect by week (Figure 2; F_(5,85) _= 10.36 *p* < .0001), indicating differential change over time in motor performance. However, there was also a significant effect by treatment (F_(3,85) _= 56.89 *p* < .0001), indicating the potential beneficial effect of leptin treatment on motor function and coordination in TDP‐43^A315T^ mice.

### 3.2 Leptin treatment altered peripheral levels of ghrelin, resistin and leptin in plasma of TDP‐43^A315T^ mice

**FIGURE 3:**
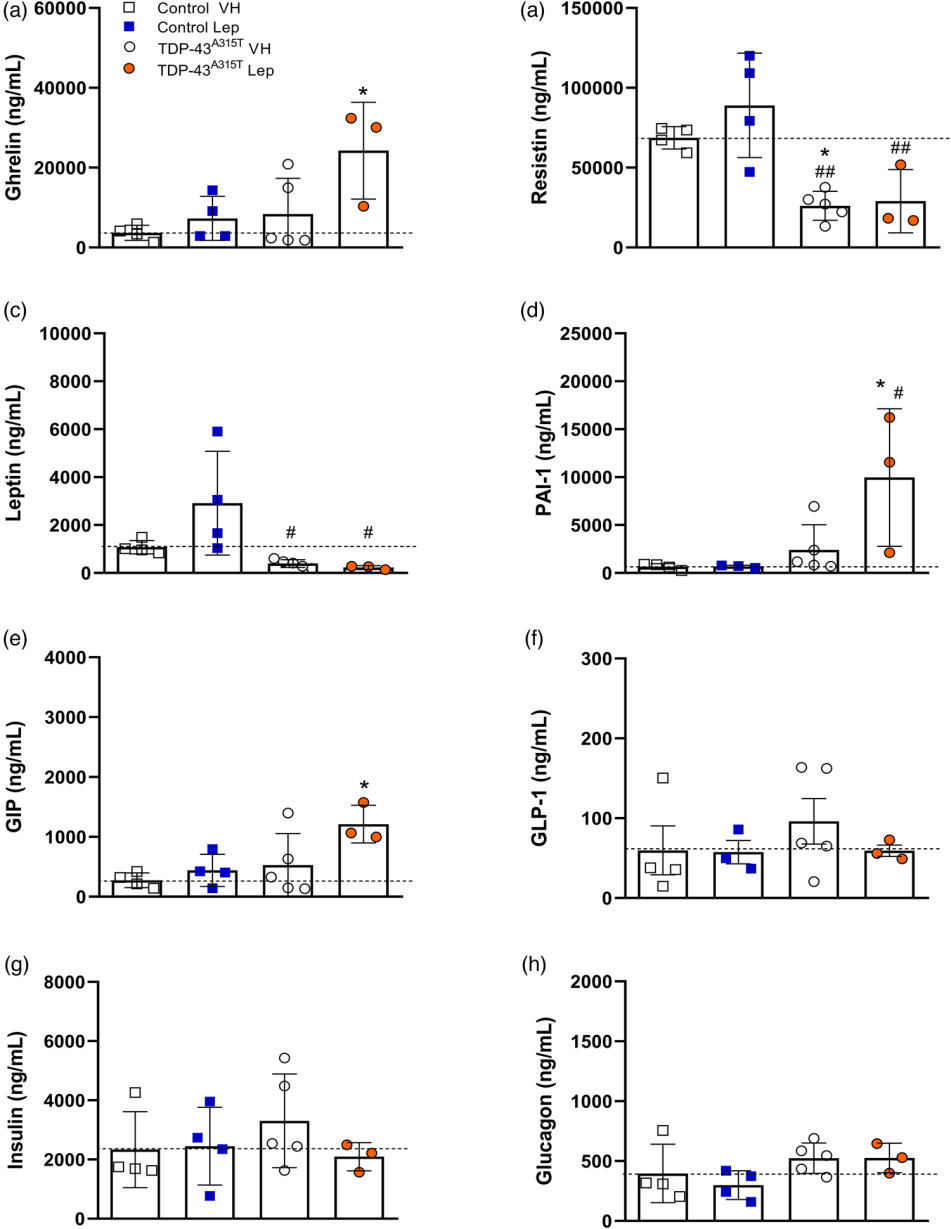
Adipocytokines and metabolic biomarkers of insulin resistance levels are altered by leptin treatment. Plasma (a) total ghrelin, the adipokines, (b) resistin, and (c) leptin, and metabolic biomarkers of insulin resistance (d) PAI‐1, (e) GIP, (f) GLP‐1, (g) insulin, and (h) glucagon) were measured in WT controls and TDP‐43^A315T^ mice treated with leptin or VH at disease end‐stage using Luminex^®^ 200™ technology. Kruskal–Wallis test was performed followed by Dunett's post hoc test to compare all groups with VH‐treated WT mice, while Bonferroni post hoc test was used for multiple comparisons between all groups, where * *p* < .05 versus VH‐treated WT mice; # *p* < .05, ## *p* < .01  versus Lep‐treated WT mice. Values are expressed as mean ± SEM for the different groups: VH‐treated WT mice (*n*  =  4, white square), Lep‐treated WT mice (*n*  =  4, blue square), VH‐treated TDP‐43^A315T^ mice (*n*  =  5, white circles), and Lep‐treated TDP‐43^A315T^ mice (*n*  =  3, orange circle)

**FIGURE 4:**
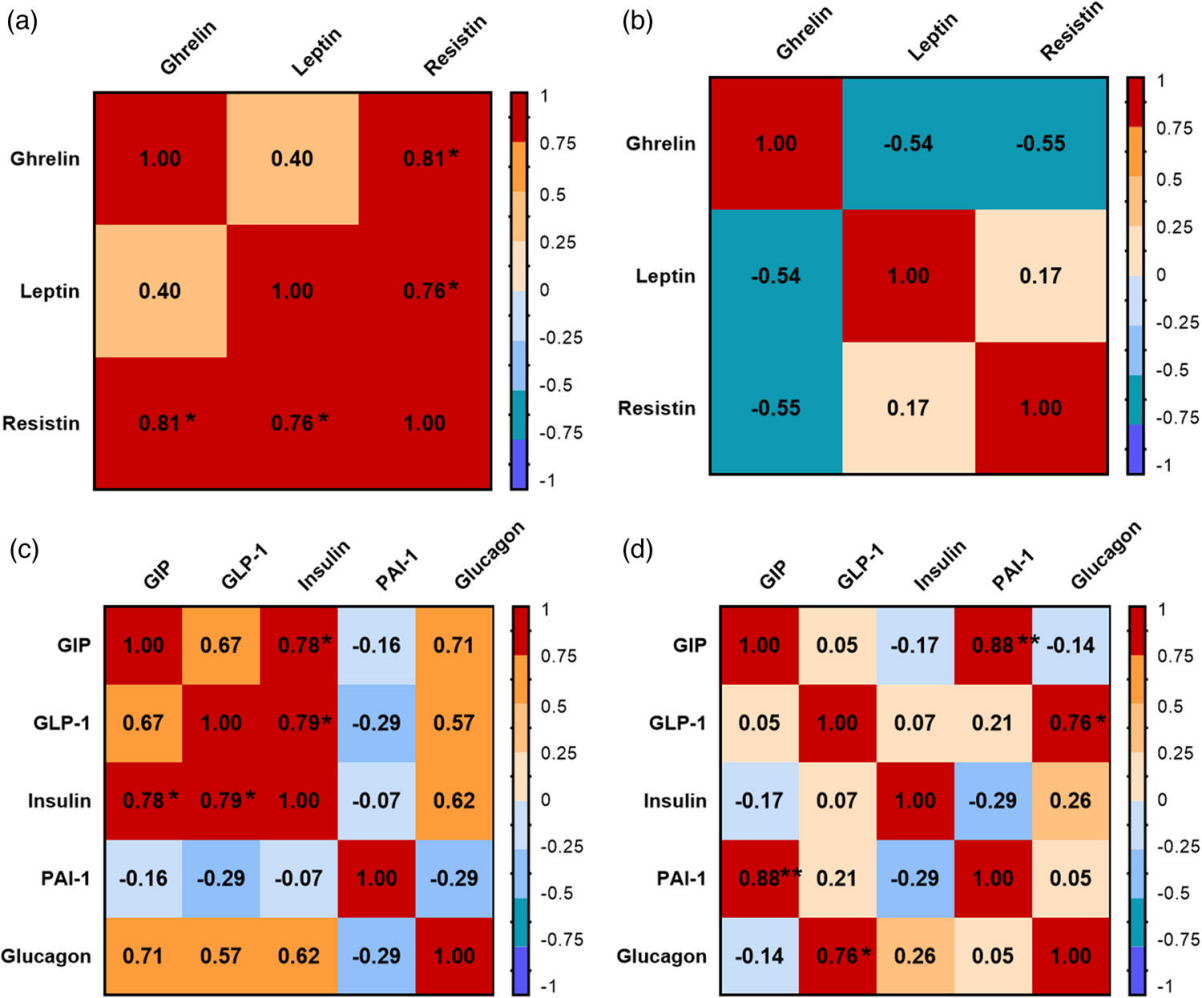
Spearman correlations for adipocytokines and metabolic biomarkers of insulin resistance. Red and blue squares refer to positive and negative correlations, respectively, with color intensity proportional to the correlation coefficient, as seen in the legend bar at the right. (a) Correlations of total ghrelin and the adipokines, resistin, and leptin, in WT mice treated with leptin and VH, where *p < .05. (b) Correlation of total ghrelin and the adipokines, resistin, and leptin, in TDP‐43^A315T^ mice treated with leptin and VH. (c) Correlations for the metabolic biomarkers studied in WT mice treated with leptin and VH, where *p < .05. (d) Correlations for the metabolic biomarkers studied in TDP‐43^A315T^ mice treated with leptin and VH, where *p < .05, **p < .01

We next studied how metabolic markers in plasma were affected by leptin treatment in TDP‐43^A315T^ mice compared to age‐matched WT littermates, as metabolic homeostasis is unbalanced in ALS patients (Ioannides et al., 2016), and this hormone is historically known for its important role in regulating body weight (Friedman, 2011). Plasma immunoassay analysis indicated circulating total ghrelin concentrations were increased in both WT and TDP‐43^A315T^ mice at the end‐stage of disease, being significantly increased in Lep‐treated TDP‐43^A315T^ mice compared to VH‐treated WT (*p* = .02; Figure 3a). Resistin and leptin levels also showed genotype‐specific differences (Figure 3b,c). Dunnett's post hoc test demonstrated significant differences in circulating levels of resistin VH‐treated TDP‐43^A315T^ mice compared to age‐matched WT littermates (*p* = 0.02; Figure 3b). In addition, circulating resistin and leptin concentrations were lower in TDP‐43^A315T^ mice compared to age‐matched WT littermates, with this reaching statistical significance at the end‐stage of disease in both groups compared to Lep‐treated WT mice (*p* = .0019 and *p* = .007, respectively; Figure 3b,c). Additionally, a positive linear correlation was found using Spearman's test among the plasmatic levels of ghrelin and leptin compared to resistin in WT mice (Figure 4a; *r* = 0.81, *p* < .05; *r* = 0.762, *p* < .05, respectively), while no linear correlation was found in any of the adipocytokines studied for the TDP‐43^A315T^ mice (Figure 4b).

Finally, to further analyze metabolism, circulating levels of PAI‐1, GIP, GLP‐1, insulin and glucagon peptides were measured in TDP‐43^A315T^ compared to age‐matched WT littermates treated with leptin or VH at the end‐stage of disease. Kruskal–Wallis test showed differences in PAI‐1 levels (Figure 3d; F_(3, 11) _= 5.177, *p* < .05), particularly an increase in Lep‐treated TDP‐43^A315T^ mice, as well as a tendency when comparing both treatments within the TDP‐43^A315T^ group. Similarly, circulating levels of GIP was altered (Figure 3e; F_(3, 11) _= 4.241, *p* < .05), showing an up‐regulation in the Lep‐treated TDP‐43^A315T^ group compared to VH‐treated WT. However, no difference was found when comparing TDP‐43^A315T^ mice treated with either leptin or VH, nor among the plasmatic levels of other metabolic proteins. Nevertheless, a positive linear correlation was found using Spearman's test among the plasmatic levels of GIP and GLP‐1 compared to insulin in WT animals (Figure 4c; *r* = 0.778, *p* < .05; *r* = 0.786, *p* < .05, respectively), as well as between GIP and PAI‐1 (Figure 4d; *r* = 0.881, *p* < .01) and GLP‐1 and glucagon (Figure 4d; *r* = 0.762, *p* < .05) in TDP‐43^A315T^ mice.

## 4 DISCUSSION

A growing body of evidence shows disturbances in energy metabolism in ALS (Blasco et al., 2020; Tefera & Borges, 2016; Tefera et al., 2021; Vandoorne et al., 2018), suggesting that targeting metabolism could represent a rational strategy to treat this disease. Mild obesity appears to improve survival in ALS patients (Paganoni et al., 2011), and obesity affects leptin, which is associated with a lower risk of developing ALS (Nagel et al., 2017). Indeed, plasma levels of leptin may be used as prognostic biomarker to ALS (Picher‐Martel et al., 2021). However, very little is known about the use of leptin as a promising drug for the treatment of this irreversible and progressive neurological condition. Here, we present evidence of how leptin can be exploited to derive therapeutic effects in ALS, providing novel insights about the link between leptin and some metabolic disturbances present in TDP‐43^A315T^ mice.

Here we report that leptin treatment had a significant effect on the regulation of the expression of certain adipocytokines and metabolic proteins in TDP‐43^A315T^ mice. This observation is of interest because metabolic abnormalities have been reported in both ALS patients (Dupuis et al., 2004) and mouse models of ALS (Giacobbo et al., 2021; Jawaid et al., 2014). Our results showed an increase in circulating plasma levels of ghrelin in Lep‐treated TDP‐43^A315T^ mice. This result is of particular interest in light of our recent data (Rodriguez et al., 2021), confirming how plasma levels of ghrelin, an appetite‐stimulating hormone, were highest in WT animals at end‐stage of disease, and although there was an increase between onset and end‐stage in TDP‐43^A315T^ mice they remained significantly lower compared to WT. This is consistent with our result showing that leptin treatment mitigates the sustained decline in body weight in TDP‐43^A315T^ mice over time. The opposite is observed in ALS patients (Lopez‐Gomez et al., 2021; Ludolph et al., 2020) as ALS causes loss of body weight. Thus, this slower body weight loss in Lep‐treated TDP‐43^A315T^ group compared to VH‐treated might contribute to a better disease progression. Nevertheless, other preclinical studies have shown motor improvement after leptin‐deficiency in a SOD1^G93A^ mouse model of ALS (Lim et al., 2014), and this contradiction suggests that the beneficial effects observed are not due only to leptin per se but to its interaction with the genetic background of the mouse model used. Furthermore, it is worth noting that SOD1‐causing mutations represent only a minor subset of ALS patients while TDP‐43 pathology is observed in the vast majority (∼80%) of both sporadic and familial ALS cases (Chen‐Plotkin et al., 2010; Pape & Grose, 2020). Thus, leptin treatment could be beneficial for the majority of ALS patients. Accordingly, our data indicated that Lep‐treated TDP‐43^A315T^ mice developed motor and coordination improvement, correlating with a delay on the disease duration. It may also be worthwhile to consider other potential disease‐relevant effects of leptin treatment in the TDP‐43^A315T^ mice, in which a progressive motor impairment has been described (Stallings et al., 2010). In addition, we also found lower circulating levels of the adipokine resistin in TDP‐43^A315T^ mice compared to controls WT animals, in contrast to clinical data, which have shown no differences in plasmatic levels of resistin between controls and ALS patients (Ngo et al., 2015). This result support previous unpublished data from our lab showing a downregulation of peripheral protein resistin levels in TDP‐43^A315T^ mice (Rodriguez et al., 2021). In addition to the marked decrease of circulating resistin concentrations, our results confirm highest circulating leptin levels in WT animals treated with leptin compared to VH‐treated WT, which was not observed in Lep‐treated TDP‐43^A315T^ mice. Indeed, in agreement with novel clinical data (Lee et al., 2021), data from our lab reported circulating plasma levels of leptin were lower in TDP‐43^A315T^ mice compared to WT mice at the end‐stage of disease (Rodriguez et al., 2021). Thus, it seems plausible that leptin treatment acts inhibiting the endogenous leptin production in TDP‐43^A315T^ mice, through mechanisms unknown, by acting systemically on the adipose tissue, the main source of leptin production (Zhang & Chua, 2017). Future experiments should try to corroborate this hypothesis. It will be interesting in future in vitro studies to determine the mechanism of leptin regulation in primary adipocytes of mutant TDP‐43.

Furthermore, we also found a significant increased plasma circulating levels of PAI‐1 and GIP in Lep‐treated TDP‐43^A315T^ mice. This result support previous in vitro results showing how leptin treatment up‐regulates the expression of *PAI‐1* gene in primary culture of vascular endothelial cells (HCAEC) (Singh et al., 2010). This observation is of interest because PAI‐1 is a metabolic protein associated with an increase in adiposity and body mass index (BMI) (Kahn et al., 2006; Ngo et al., 2015). The majority of ALS patients have low BMI (Dardiotis et al., 2018), and a positive correlation of plasma leptin and BMI was observed in human ALS (Ngo et al., 2015). Thus, it is plausible that the fact that circulating endogenous leptin levels are decreased in Lep‐treated TDP‐43^A315T^ mice might indicate that this effect could partly due to the exogenous leptin administration. Regarding circulating levels of GIP, low levels of this protein have been reported in ALS patients (Ferri & Coccurello, 2017). This protein stimulates insulin secretion in response to food intake (Elliott et al., 1993), and it has been proposed as an obesity‐promoting hormone (Holst & Rosenkilde, 2020). Thus, it is conceivable that the up‐regulation of circulating levels of GIP in Lep‐treated TDP‐43^A315T^ mice would help to reduce disturbances in energy metabolism associated with the progression of ALS in TDP‐43^A315T^ mice (Shan et al., 2010; Wang et al., 2013). However, future experiments should try to corroborate this hypothesis.

Finally, a positive linear correlation was found among ghrelin and leptin relative to resistin levels in the WT animals, which is related to insulin resistance. Likewise, a positive correlation was found among the plasma levels of GIP and GLP‐1 relative to insulin concentrations in the WT genotype, consistent with their role as incretins (Seino et al., 2010). However, no correlations were determined in the TDP‐43^A315T^ genotype, indicating a possible development of insulin resistance in this Tg mouse model of ALS. It should be noted that a positive correlation was found between levels of GIP and PAI‐1 as well as between GLP‐1 and glucagon in TDP‐43^A315T^ mice.

## 5 CONCLUSIONS

In summary, our research provides the first preliminary experimental evidence for a potential therapeutic effect of leptin treatment in motor function as well as in some metabolic disturbances present in TDP‐43^A315T^ mice. Comparatively the disease duration was longer in Lep‐TDP‐43^A315T^ mice. Our laboratory is currently carrying out studies to determine the main role of leptin at the biological level in TDP‐43‐related disease progression. In this context, our scientific investigations have recently discovered how leptin plays an important role in ALS. Our lab has provided the first experimental evidence suggesting that ALS may be associated with alterations in leptin signaling pathways that might result in a leptin resistant state, and that this could play a critical role in the characteristic irreversible and progressive pathological changes associated with ALS (Ferrer‐Donato et al., 2021). However, one caveat of this study that should be take into consideration is that this preliminary in vivo new study conducted in TDP‐43^A315T^ mice only evaluated the impact of leptin treatment during the asymptomatic stage of the disease, and only during two consecutive weeks, rather than analyzing the effect of the exogenous leptin administration until the end‐stage of disease, or beginning after disease onset. Therefore, further mechanistic studies, with variable leptin concentrations and time‐treatment duration, may require larger sample sizes at defined time‐points. Determining the role of leptin and its mechanistic actions may provide a new avenue for therapeutic development for this fatal condition.

## AUTHOR CONTRIBUTION

A.F.‐D.: Methodology; investigation. A.C.: Formal analysis; writing—original draft preparation; writing—review and editing. P.F.: Methodology; formal analysis; C.M.F.‐M.: Conceptualization; methodology; formal analysis; investigation; writing—review and editing; supervision; project administration; funding acquisition. All authors have read and agreed to the published version of the manuscript.

## CONFLICT OF INTEREST

The authors declare no conflict of interest.

### PEER REVIEW

The peer review history for this article is available at https://publons.com/publon/10.1002/brb3.2465

## Data Availability

The data that support the findings of this study is available from the corresponding author upon reasonable request.
